# Two new species of *Meropidia* Hippa & Thompson, 1983 (Diptera, Syrphidae) from the Andes Mountains

**DOI:** 10.3897/zookeys.338.6093

**Published:** 2013-10-02

**Authors:** Mírian N. Morales, Gunilla Ståhls, Heikki Hippa

**Affiliations:** 1 Finnish Museum of Natural History, PO Box 17, FI–00014 University of Helsinki, Finland Universidade Federal de Lavras Lavras Brazil; 2 Gribbylunds allé 2, SE–183 65 Täby, Sweden Swedish Museum of Natural History Sweden

**Keywords:** Description, Eristalinae, *Meropidia*, identification key, Neotropical region, taxonomy

## Abstract

Two new species of *Meropidia* Hippa & Thompson, 1983 (Diptera, Syrphidae) are described, *Meropidia nitida* Morales, **sp. n.** and *Meropidia flavens* Hippa & Ståhls **sp. n.**, from Bolivia and Colombia respectively. A key to all described *Meropidia* species is provided.

## Introduction

The Neotropical region has a very high diversity of flower flies (Diptera, Syrphidae), comprising just over 30% of the currently recognized species in the world (Thompson et al. 2010) and many species new to science have continuously been described for genera confined to this region (e.g. Rotheray et al. 2007, 2009, Carvalho Filho and Esposito 2009, Morales et al. 2009, Ricarte et al. 2012). However, few studies on regional Syrphidae fauna in the Neotropical region exist, particularly on South America (see Thompson and Marinoni 2003, Montoya et al. 2012 for a review).

The genus *Meropidia* was described by Hippa and Thompson in 1983, comprising three species: *Meropidia nigropilosa* Thompson, *Meropidia neurostigma* Hippa and *Meropidia rufa* Thompson. All described species are confined to the Neotropical region, specifically occurring on Tropical Andes (Hippa and Thompson 1983, Montoya et al. 2012).

*Meropidia* Hippa & Thompson, 1983 belongs to the subfamily Eristalinae and was placed in the tribe Milesiini, subtribe Tropidiina (Hippa and Thompson 1983). This subtribe comprises seven genera in addition to *Meropidia*: *Calcaretropidia* Keiser, 1971, *Macrozelima* Stackelberg, 1930, *Nepenthosyrphus* Meijere, 1932, *Orthoprosopa* Macquart, 1850, *Senogaster* Macquart, 1843, *Syritta* Lepeletier & Serville, 1828 and *Tropidia* (Meigen, 1822). Of these genera only *Meropidia* and *Senogaster* are confined to the Neotropical region, while species of *Syritta* and *Tropidia* also occur in the region (Thompson 2013). These genera can be readily distinguished from *Meropidia* by a swollen metafemur, often with armature, as *Meropidia* has a slightly thickened throughout and slightly arcuate metafemur, with no modifications (Thompson 1972).

Besides the simple metafemur, *Meropidia* is recognized as a moderately pilose taxa, with sexually dimorphic face (male with a broad, low, medial tubercle and female concave), with pollinose pattern on mesonotum and pilose metasternum. It has eye bare and male is very narrowly dichoptic. The wing has variable numbers of stigmatic crossveins; cell r1 open; vein R4+5 slightly sinuate, with a short petiole; vein r-m strongly oblique and long, ending at outer ¼ of discal cell, vein A1+CuA2 very long. The male sex is known only for one species, *Meropidia neurostigma* (Hippa and Thompson 1983). The authors of this genus suggest that *Meropidia* is sister group of *Orthoprosopa* and *Paratropidia* Hull, 1949, being these three taxa the plesiomorphic sister group to the other genera of Tropidiina (Hippa and Thompson 1983).

The most appropriate identification key to run for the genus *Meropidia* is that published in Thompson (1999). Because *Meropidia* is a small genus with species confined to a very particular region, there is no information about biology of its species, neither about immature stages. In addition to the original description work (Hippa and Thompson 1983) and the identification key for Neotropical genera (Thompson 1999), *Meropidia* is also cited by Montoya et al. (2012), where is presented a review for flower flies fauna of Colombia and a comprehensive review of the literature concerning Colombia and the Neotropical region. It is noteworthy that in a previous work presenting a faunal list of Colombia, Gutierrez et al. (2005) had omitted the genus *Meropidia* from that list, but Montoya et al. (2012) included the omitted species *Meropidia neurostigma* Hippa, 1983 and recorded *Meropidia nigropilosa* Thompson, 1983 as new to Colombia.

No additional species have been described for *Meropidia* since 1983, and in the present work two new species, *Meropidia nitida* Morales, sp. n. and *Meropidia flavens* Hippa & Ståhls, sp. n., from Bolivia and Colombia respectively, are described.

## Material and methods

Terminology follows Thompson (1999). The identification key was constructed based on the key from Hippa and Thompson (1983).

Type localities and holotype holding institutions are specified for each species. Location and identifications labels are indicated with quotation marks (“ ”), and which line on the label separated by a forward slash (/). Handwritten information on labels is indicated in italics. The acronyms used for collections follow the standard of the *Systema Dipterorum* (Thompson 2013), and their equivalents are listed below:

AMNH American Museum of Natural History, New York, USA.


MNHN Muséum National d’Histoire Naturelle, Paris, France.


All measurements are in millimeters and were taken using a reticule in a Nikon SMZ1000 stereomicroscope. Photographs of all new species were provided and were composed using the Combine ZP software based on images of pinned specimens.

In addition, were also provided photographs of the *Meropidia nigropilosa* holotype, which is deposited at Natural History Museum, London, UK, and has no images available in an online source.

## Description of new species

The species described here do not have a concave face, neither a wholly pollinose face as defined in Hippa and Thompson 1983 as diagnostic characters for *Meropidia* females; but, it is flat with a sub-medial and sub-developed tubercle, and shiny medially. A full generic description of *Meropidia* is not necessary here, but it is necessary to record that concavity and pollinosity of female face are not diagnostic characters for the genus *Meropidia*. Otherwise we have no additions or changes to the generic description.

### Meropidia nitida

Morales sp. n.

F2BF479B-6312-327A-01CA-2C1791547740

http://zoobank.org/791B17B7-CC3C-4413-A750-8EDD936EBF7C

http://species-id.net/wiki/Meropidia_nitida

Figs 1–8

#### Type specimens.

HOLOTYPE. Adult female, missing left midleg. Deposited at AMNH, New York City, USA. Type locality: BOLIVIA. Labels (Figs 6–8) “BOLIVIA: Sta Cruz Dept. / Caballero Prov., PN Amboró / 17°50.124'S, 64°23.454'W / 2070 m, X.18–19.2001 / S. Spector & J. Ledezma / mallaise ‘sic’ trap”; “COBIMI0011824”; “HOLOTYPE / *Meropidia nitida*/ Morales, 2013”.

**Paratype.** Adult female. BOLIVIA. Deposited at AMNH, New York City, USA. Labels: same data as Holotype; “COBIMI0011826”; “PARATYPE / *Meropidia nitida*/ Morales, 2013”.

#### Description.

Adult female. Body size: 9–10.5 mm

**Figures 1–8.**
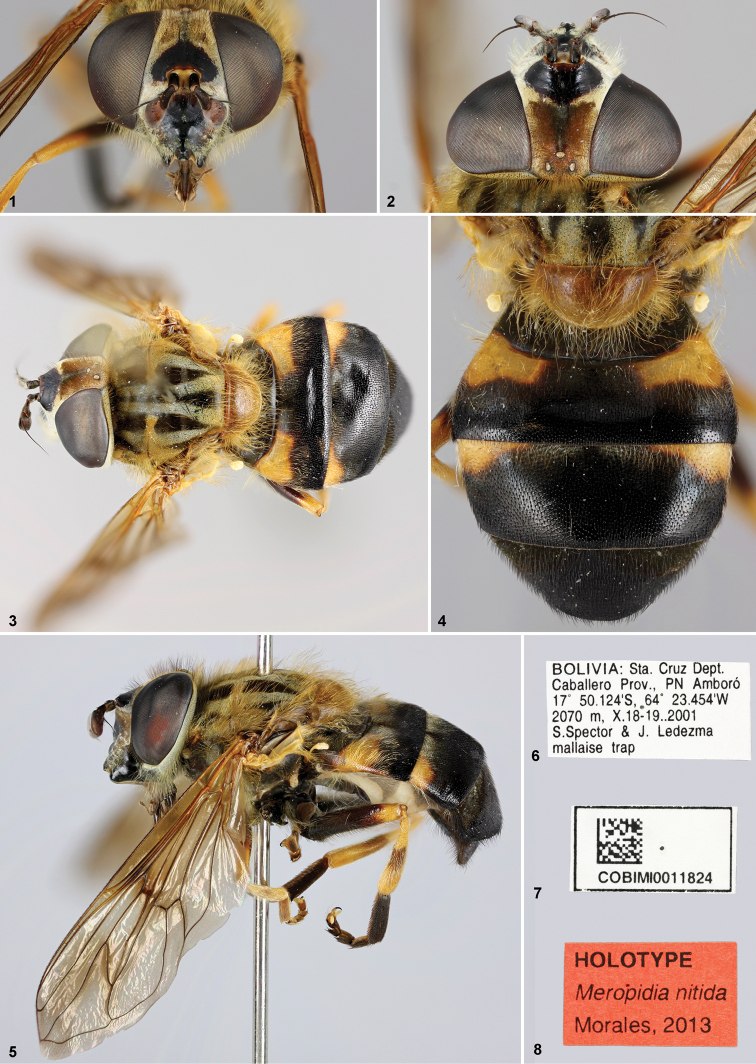
*Meropidia nitida* Morales, sp. n.,Holotype. **1** head, anterior view **2** head, dorsal view **3** habitus dorsal **4** abdomen, dorsal view **5** habitus lateral **6–8** labels.

**Head**. Face shiny black medially, becoming brownish to yellowish laterally, white pollinose laterally to bases of antenna, yellow pilose on pollinose area (Figs 1, 5), with a subdeveloped tubercle on inferior half (Fig. 5); gena shiny black, white pollinose and yellow pilose posteriorly (Fig. 5); facial stripes brown pollinose, with short yellow pile; lunula dark brown; frons shiny black in an anterior triangular area (Figs 1, 2), with brownish pile which became yellowish on their apexes, yellow pollinose elsewhere, sparse medially, intermixed with brownish and yellow pile; ocelli yellowish; ocellar triangle slightly isosceles obtuse (Fig. 2); occiput dark, yellowish pollinose dorsally, white pollinose elsewhere (Fig. 5), brownish and yellowish pilose dorsally, light yellow pilose elsewhere. *Antenna*. Scape and pedicel 2× longer than broad, dark brown (Fig. 1); arista dark brown, except apex reddish, with very short vestiture, downy; distance between antenna approximately 1.5 times its basal diameter (Figs 1, 2).

**Thorax**. Postpronotum yellow, densely pale pollinose and yellow pilose; scutum black, golden pollinose on lateral margins and on transverse suture, four longitudinal pale pollinose vittae extending in front of scutellum (Fig. 3), long yellow pilose (Fig. 5); scutellum yellowish, with longer yellow pile than scutum (Figs 3, 4), with scattered brownish pile on margin. Postalar callus with longer pile than scutum pile, as long as in scutellum, anterodorsal edge with long pile on posterior half, postalar wall bare. Calypter, haltere, plumula and spiracles yellow. Pleura dark brown, sparsely pale pollinose, except densely pollinose on posterior anepisternum, dorsal part of katepisternum and anterior anepimeron. Proepimerum, posterior anepisternum, anterior anepimeron and katatergum yellow pilose; pile of posterior anepisternum and anterior anepimeron somewhat produced posteriorly. Katepisternum mostly yellow pilose, except a few dark brown pile ventrally; metasternum black pilose.

*Legs*: simple, coxae and trochanters dark brown, mostly yellow pilose, intermixed with brownish pile. Pro and mesofemora brownish on basal half and yellow on apical half, yellow pilose, with longer pile ventrally; metafemur mostly dark brown, but apically yellow (Fig. 5), yellow pilose, ventral pile shorter than anterior and posterior surfaces, but longer than dorsal surface, longest pile on anterior and posterior surfaces erect, tilted apically elsewhere. Tibiae yellow, except metatibia slightly brownish medially (Fig. 5), yellow pilose. Metatarsus dark brown (Fig. 5), mostly brownish pilose, with few scattered yellow pile on anterior and posterior surfaces; pro and mesotarsus brown, lighter than metatarsus, yellow pilose.

*Wing*: completely microtrichose; vein CuP long, almost reaching the level of posterior apex of cell bm (Fig. 5); alula shorter than anal lobe;

**Abdomen.** Tergum I black (Figs 3, 4), lateral corners brownish yellow, with long yellow erect pile laterally, shortest black tilted backward pile elsewhere; tergum II mostly black, with yellow pile on the yellow maculae region (Figs 3, 4), laterally they are erect and longer, on anteromedial 1/3 yellow pilosity tilted backward, pile black and tilted backward elsewhere, scattered pale pollinose on anterior half; tergum III mostly black, with yellow pile on lateral corners and on the yellow macula region (Figs 3, 4), black, posteriorly inclined pile elsewhere, whitish pollinose on anterior half; tergum IV black (Figs 3, 4), black pilose, except white pilose on anterior corners, whitish pollinosity forming two anterior triangular–like macula. Sterna dark brown, slightly pale pollinose; sterna I–III yellow pilose, IV yellow pilose intermixed with brownish pile; sternum V brownish pilose.

#### Comments.

The holotype has four stigmatic crossveins on the left wing and five on the right; in the paratype there are six stigmatic crossveins on both wings. Only type material is known.

### Meropidia flavens

Hippa & Ståhls sp. n.

833E2855-7883-5A67-9915-5B14D8558E1C

http://zoobank.org/5B54C6D3-4E88-4DD3-A585-810EB9DED488

http://species-id.net/wiki/Meropidia_flavens

Figs 9–15

#### Type specimen.

HOLOTYPE. Adult female, in good condition, except arista missing. Postabdominal segments V and beyond removed and placed in plastic vial on the same pin as the specimen. Deposited at NMNH, Paris, France. Type locality: COLOMBIA. Labels (Figs 13–15) “*Bogota* / *October* / *1892*”; “*This lable* ‘sic’/ *made by* / *Hippa* / *1984*”; “HOLOTYPE / *Meropidia flavens* / Hippa & Ståhls, 2013”.

#### Description.

Adult female. Body size: 11 mm

**Figures 9–15.**
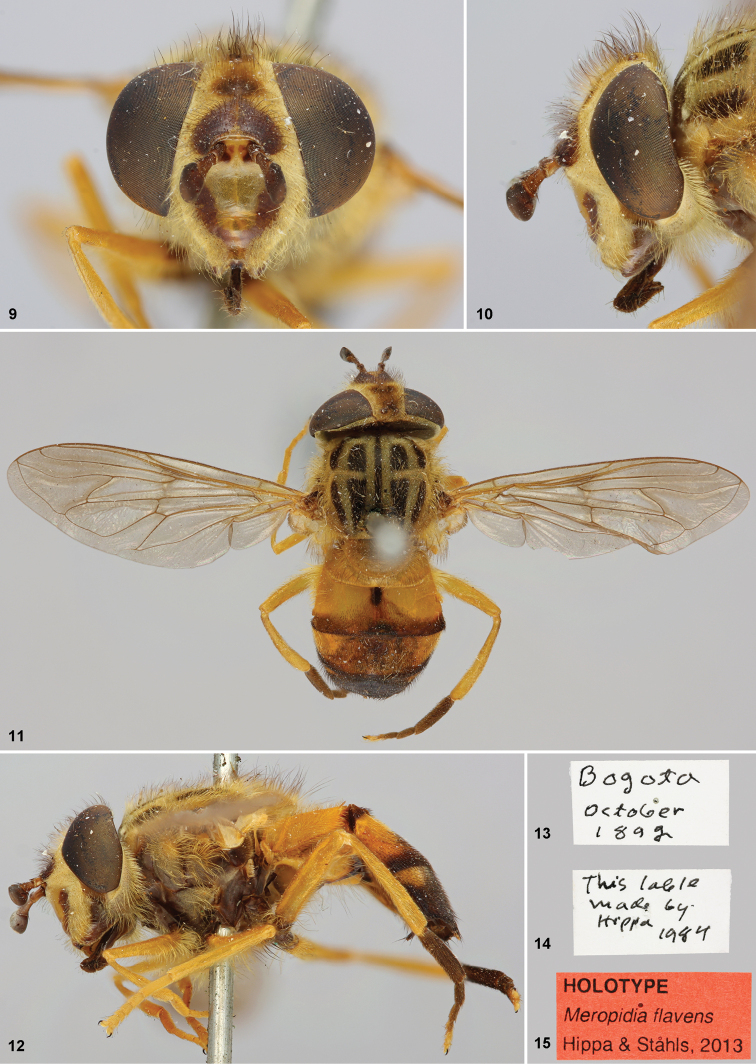
*Meropidia flavens* Hippa & Ståhls, sp. n., Holotype **9** head, anterior view **10** head, lateral vie **11** habitus dorsa **12** habitus lateral **13–15** labels.

**Head.** Elliptical in frontal view, (Fig. 11); face with a subdeveloped tubercle on inferior half (Fig. 10), shiny yellow medially, with brown U-shaped shiny area surrounding tubercle (Fig. 9), yellowish pollinose elsewhere, yellow pilose on pollinose areas; gena densely pale pollinose and long yellow pilose (Fig. 10); facial stripes brownish, short, matte, pale pollinose and pilose; lunula yellow, frontal area above lunula dark brown (Fig. 9), bare, brownish pilose; frons broad, densely yellowish pollinose and long pale pilose, antero-medial roundish and ocellar area bare; ocellar triangle slightly isosceles obtuse (Fig. 11), brownish long pilose; occiput densely pale pollinose, pale pilose, with some short black pile. *Antenna*: Scape brownish, 2× longer than broad, short dark pilose dorsally; pedicel brownish about 2.5–3× longer than broad, with short dark pile dorsally and ventrally; basoflagellomere darkbrown, shorter than high (Figs 9, 10); distance between antenna approximately twice its basal diameter (Fig. 9).

**Thorax**. Postpronotum yellow, densely pale pollinose and yellow pilose; scutum blackish, with four broad, longitudinal pale pollinose vittae extending to transverse pollinose area in front of scutellum (Fig. 11), long pale pilose, pale pollinose around transverse suture; anterodorsal edge of postalar callus with long pile on posterior half; scutellum yellowish (Fig. 11), long pale pilose, margin with pale pilosity with apex becoming browner, pile as long as scutellum. Calypters yellow with yellow marginal pilosity. Haltere yellow. Pleura brownish, slightly pollinose, except densely pale pollinose on posterior anepisternum, dorsal katepisternum and katepimeron. Propleura, posterior anepisternum, dorsal and ventral katepisternum, anepimeron with long yellow pilosity, katatergum with shorter yellow pilosity; metasternum black pilose.

*Legs*: simple, metafemur straight; yellow, except for coxae and trochanters brown (Fig. 12), femora narrowly brown antero-basally, and metatarsal segments 1–4 dark brown (Figs 11, 12).

*Wing*:completely microtrichose; slightly brownish along veins, wing veins brownish-yellow; four stigmatic crossveins; vein CuP long, almost reaching the level of posterior apex of cell bm (Fig. 11); alula shorter than anal lobe.

**Abdomen**. Tergum I yellow (Fig. 11), yellow pilose anteriorly, black short pilose posteriorly; tergum II yellow with brown spot antero-medially and posterior brownish stripe (Fig. 11), with long yellow pile at anterior corners and triangular area with short black pile; tergum III with antero-lateral corners broadly yellow (Fig. 11) and with yellow pile, with posterior brownish stripe with short black pile; tergum IV brown with antero-lateral corners yellow (Fig. 12) with short black pile. Sterna brownish, long pale pilose; sternum II with lateral areas yellow.

#### Comments.

Only type known.

### Identification key for the *Meropidia* species

**Table:** 

1	First tergum black (Figs 4, 16), at least yellowish to orange on posterior margin; metafemur mostly black, at least on basal 1/5	2
–	First tergum and femora entirely yellow or orange	4
2	Pro and mesofemora orange (Fig. 17); metafemur very narrowly black in basal 1/5 or less; metabasitarsus partially black (Fig. 17); mesonotum and scutellum with extensive black pile; (Colombia)	*Meropidia nigropilosa* Thompson
–	Pro and mesofemora black on basal half and yellow on apical half; mesonotum and scutellum with extensive yellow pile	3
3	Face shiny black medially (Fig. 1); frons shiny black in an anterior triangular area (Figs 1, 2); metatibia slightly brownish medially (Fig. 5); (Bolivia)	*Meropidia nitida* Morales, sp. n.
–	Face and frons (or frontal triangle) yellowish orange, extensively yellow pollinose; metatibia entirely yellow, without markings; (Bolivia)	*Meropidia neurostigma* Hippa
4	Face shiny yellow medially, with a brown U-shaped shiny area surrounding tubercle (Fig. 9); scutum with broad and longitudinal black markings (Fig. 11); metatarsal segments 1–4 dark brown (Figs 11, 12); (Colombia)	*Meropidia flavens* Hippa & Ståhls, sp. n.
–	Face orange, without dark brown markings surrounding tubercle; scutum with very narrow longitudinal black markings; metatarsal segments orange; (Ecuador)	*Meropidia rufa* Thompson

**Figures 16–17.**
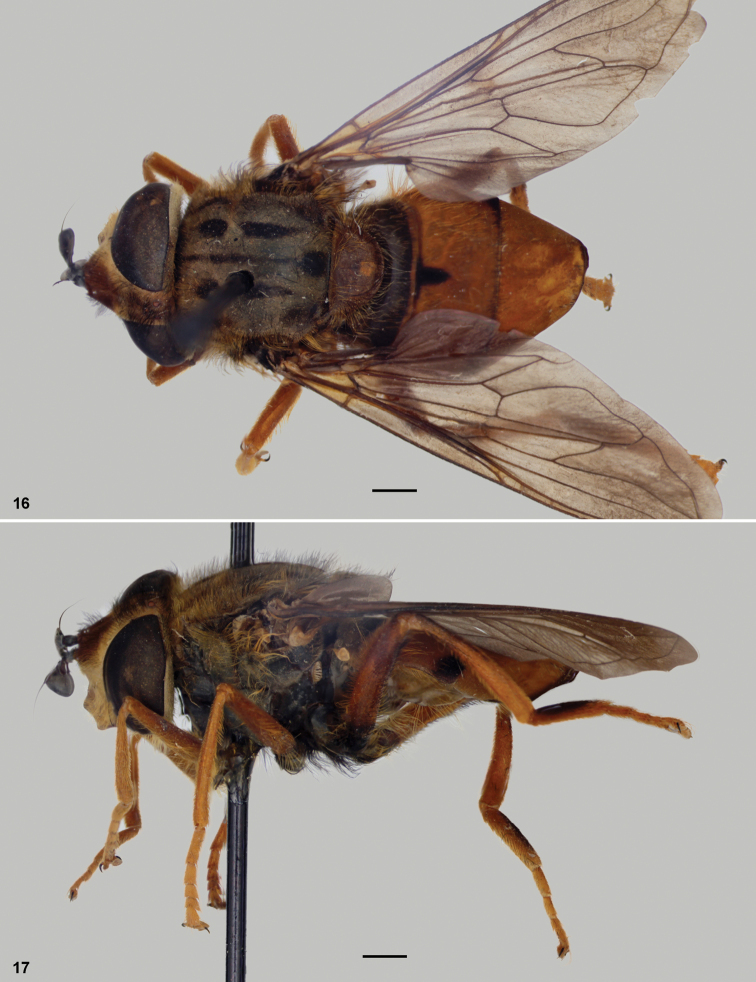
*Meropidia nigropilosa* Thompson. Holotype deposited at Natural History Museum, London, UK **16** habitus dorsal **17** habitus lateral. Scale bar: 1mm.

## Discussion

The Tropical Andes, including parts of Venezuela, Colombia, Ecuador, Peru, Bolivia and Argentina, is the biologically richest and most diverse of Earth’s biodiversity hotspots areas, mainly for vertebrates and plants (Myers et al. 2000). Although that area retains about 25% of its primary vegetation, is believed to contain, at least, 20,000 known plant endemics and probably thousands of species remain to be discovered there (Myers et al. 2000). Because many insects are associated with plants, the extreme plant endemism of the Tropical Andes suggests a similar high level of insect diversity and endemism (Larsen et al. 2011).

The high altitudes of the Andes (above 3000 masl) include the most endangered and vulnerable ecosystem in South America and it is one of the three areas where the largest changes in fauna are predicted as a result of climate change (e.g. Lawler et al. 2009, Larsen et al. 2011).

Montoya et al. (2012) found that the Colombian Andes has the highest diversity and number of endemic species of Syrphidae. Species in the Neotropical genera *Macrometopia* Philippi, 1865, *Meropidia*, *Talahua* Fluke, 1943 and *Tuberculanostoma* Fluke, 1943 were in their study found to be restricted to the high altitudes above 3000 masl of the Colombian Andes.

Therefore, these taxa might be considered focus groups in future conservation projects due to the predictions of the impact of the climate change. The species described here also occur at altitudes between 2000–3000 masl and are thus important additions to the knowledge of the biodiversity of the high Andes flower fly fauna.

Of the five *Meropidia* species now known for the science, the male sex is still only known for *Meropidia neurostigma*. The hitherto described *Meropidia* species are distributed in Bolivia, Colombia and Ecuador.

## Supplementary Material

XML Treatment for Meropidia nitida

XML Treatment for Meropidia flavens
